# Polystyrene-Based Hydroxide-Ion-Conducting Ionomer: Binder Characteristics and Performance in Anion-Exchange Membrane Fuel Cells

**DOI:** 10.3390/polym13050690

**Published:** 2021-02-25

**Authors:** Ji Eon Chae, So Young Lee, Sung Jong Yoo, Jin Young Kim, Jong Hyun Jang, Hee-Young Park, Hyun Seo Park, Bora Seo, Dirk Henkensmeier, Kwang Ho Song, Hyoung-Juhn Kim

**Affiliations:** 1 Center for Hydrogen and Fuel Cell Research, Korea Institute of Science and Technology (KIST), Hwarang-ro 14-gil 5, Seongbuk-gu, Seoul 02792, Korea; 218016@kist.re.kr (J.E.C.); sylee5406@kist.re.kr (S.Y.L.); ysj@kist.re.kr (S.J.Y.); jinykim@kist.re.kr (J.Y.K.); jhjang@kist.re.kr (J.H.J.); parkhy@kist.re.kr (H.-Y.P.); hspark@kist.re.kr (H.S.P.); brseo@kist.re.kr (B.S.); henkensmeier@kist.re.kr (D.H.); 2 Department of Chemical and Biological Engineering, Korea University, Anam-ro 145, Seongbuk-gu, Seoul 02841, Korea

**Keywords:** anion-exchange membrane fuel cell, polystyrene, electrode binder, anion-exchange ionomer, cell flooding, water management

## Abstract

Polystyrene-based polymers with variable molecular weights are prepared by radical polymerization of styrene. Polystyrene is grafted with bromo-alkyl chains of different lengths through Friedel–Crafts acylation and quaternized to afford a series of hydroxide-ion-conducting ionomers for the catalyst binder for the membrane electrode assembly in anion-exchange membrane fuel cells (AEMFCs). Structural analyses reveal that the molecular weight of the polystyrene backbone ranges from 10,000 to 63,000 g mol^−1^, while the ion exchange capacity of quaternary-ammonium-group-bearing ionomers ranges from 1.44 to 1.74 mmol g^−1^. The performance of AEMFCs constructed using the prepared electrode ionomers is affected by several ionomer properties, and a maximal power density of 407 mW cm^−2^ and a durability exceeding that of a reference cell with a commercially available ionomer are achieved under optimal conditions. Thus, the developed approach is concluded to be well suited for the fabrication of next-generation electrode ionomers for high-performance AEMFCs.

## 1. Introduction

In view of the growing importance of energy depletion and environmental problems, highly efficient eco-friendly fuel cells have attracted much attention as next-generation energy sources, providing an alternative to fossil fuels. Polymer electrolyte fuel cells such as proton-exchange membrane fuel cells (PEMFCs) and anion-exchange membrane fuel cells (AEMFCs) are classified according to the ion exchanged in the polymer electrolyte membrane [1]. PEMFCs are the most commercialized and are used to generate without limits on placement, including in houses and buildings, transportation systems, and portable devices [2,3]. In addition, microbial fuel cells using proton exchange membranes have attracted attention for their improved performance owing to the development of anode materials [4,5]. Meanwhile, AEMFCs contain hydrocarbon polymer electrolyte membranes and are operated at low temperatures of ≤ 100 °C [6]. As the oxygen reduction overpotential in alkaline environments is lower than that in acidic ones, fuel cells operated under alkaline conditions offer the advantage of using low-cost non-precious-metal-based oxygen reduction catalysts [7]. Therefore, as the use of AEMFCs can significantly reduce the overall system cost, oxygen reduction catalysts equivalent to Pt [8,9] and new polymeric materials [10,11,12,13] for anion-exchange membranes (AEMs), which are of key importance for AEMFC durability, are highly sought after. 

AEMs feature cationic groups (e.g., quaternary ammonium, imidazolium, phosphonium, piperidinium, quinuclidinium, and guanidinium) attached to a hydrocarbon polymer backbone as hydroxide ion carriers and exhibit low gas permeability, high hydroxide-ion conductivity, good mechanical properties, and high chemical stability/durability under alkaline conditions [14,15,16,17,18,19] In addition to AEMs, another important polymeric component of AEMFCs is the electrode ionomer, which is the core material of the membrane electrode assembly (MEA) and acts as a physical binder to uniformly disperse the catalyst and fix the catalyst layer and the AEM. Moreover, the ionomer also acts as a pathway for hydroxide ion transfer in the catalyst layers, and it exhibits properties that are sometimes very different from those of AEMs. 

In particular, the electrode ionomer significantly influences catalyst layer morphology and fuel cell performance [4,5,20,21], e.g., the free volume of the polymer structure and the permeability of the electrode to gases can both be increased by using electrode ionomers highly permeable to fuels [20,21]. Choi et al. studied the effect of spirobiindane units of bulky moieties in poly(ether sulfone)s, showing that the high gas permeability of the electrode binder contributes to AEMFC performance improvement [20], whereas Liang et al. introduced an ionomer cross-linking immobilization strategy to realize efficient gas permeation and prevent the coalescence of Pt/C nanoparticles [21].

Another important issue not covered by AEMs is the problem of phenyl group adsorption to the catalyst, i.e., the phenyl group is believed to block the hydrogen oxidation reaction (HOR) active sites of Pt [22]. This problem can be solved by either using non-Pt catalysts or minimizing ionomer adsorption to Pt catalysts. Maurya et al. characterized non-Pt HOR catalysts with different surface adsorption properties using rotating disk electrode measurements, density functional theory calculations, and fuel cell performance tests [23]. Kim et al. studied the effects of introducing a benzyltrimethylammonium hydroxide side chain into aryl-ether-free polyphenylene and the alkyl chain of its main chain on phenyl adsorption to Pt-based HOR catalysts [24]. Their results showed that AEMFC performance can be enhanced through the use of ionomers with fewer phenyl moieties or with phenyl moieties located near bulky cationic functional groups. Thus, the advances in AEMFC development can be ascribed to the design of electrode ionomers with on-demand properties.

Friedel–Crafts acylation and reduction have been applied by several groups to attach functional groups to the benzene rings of polymer backbones such as poly(phenylene), polystyrene-b-poly(ethylene-co-butylene)-b-polystyrene, poly(phenylene oxide), and poly(aryl ether) [25,26,27,28,29]. Chu et al. used a polystyrene (PS)-based electrode binder with an alkylammonium-group-bearing backbone as an in-house-made ionomer for poly(phenylene oxide)-based membranes [30]. However, no specific structural analysis or explanation of PS-based ionomer has been reported so far. Herein, we prepared a PS-based electrode ionomer through radical polymerization followed by Friedel–Crafts acylation, reduction, and quaternization. Our results revealed that the aryl-ether-free backbone can secure stability in an alkaline environment, and we further probed the effects of the polymer molecular weight and length of the quaternary-ammonium-group-bearing side chains on ionomer performance. As a result, the developed strategy was found to hold great promise for the facile synthesis of electrode ionomers for high-performance AEMFCs.

## 2. Materials and Methods

### 2.1. Materials

Benzenesulfonyl chloride (99%), styrene (99%), 2,2′-azobis(2-methylpropionitrile) (AIBN; 0.2 M in toluene), activated basic alumina, AlCl3 (99%), 6-bromohexanoyl chloride (97%), 3-bromopropionyl chloride (technical grade), trifluoroacetic acid (97%), triethylsilane (98%), 45 wt% trimethylamine in H_2_O, anhydrous toluene, 1,2-dichloroethane, *N,N*-dimethylformamide (DMF), and *N*-methyl-2-pyrrolidone (NMP for HPLC) were obtained from Aldrich and used without further purification. KOH was obtained from Daejung Chemicals Co.

### 2.2. Synthesis of PS-Based Ionomers

#### 2.2.1. Synthesis of the PS Backbone

Styrene was subjected to free radical polymerization using benzenesulfonyl chloride as a chain transfer reagent and AIBN as an initiator [31]. Prior to polymerization, styrene was passed through a basic alumina column to remove the polymerization inhibitor. A 100-mL Schlenk tube was charged with benzenesulfonyl chloride (0.20 g, 1.13 mmol), AIBN solution (0.54 mL, 0.11 mmol), toluene (10 mL), and styrene (7.74 g, 74.35 mmol), sealed with a rubber septum, degassed by five-fold freeze–thaw cycling, and filled with Ar. After polymerization, which was performed at 60 °C for 24 h, the mixture was poured into a large amount of methanol, and the white precipitate was washed several times with methanol and dried in a vacuum oven at 80 °C to afford PS in 22.6% yield. The number average molecular weight (*M*_n_) and polydispersity index (PDI) of PS were determined as 9735 g mol^−1^ and 1.56, respectively. PS with different molecular weights was prepared by adjusting the styrene:benzenesulfonyl chloride molar ratio (65–1000:1).

#### 2.2.2. Synthesis of Bromoacylated PS (SxxBACyy-Cn)

The obtained PS (1 g, *M*_n_ = 9735 g mol^−1^, PDI = 1.56) was dissolved in 1,2-dichloroethane (15 mL) contained in a 250-mL round-bottom flask equipped with a condenser and a magnetic stirrer. The solution was supplemented with AlCl_3_ (0.41 g, 3.08 mmol) and 6-bromohexanoyl chloride (0.66 g, 3.08 mmol) and stirred at room temperature. After 3 h, the deep orange mixture was poured into a large amount of methanol, and the precipitate was washed several times with the same and dried in a vacuum oven at 80 °C to afford bromoacylated PS in 85% yield. The obtained samples are hereafter denoted by SxxBACyy-Cn, where Sxx stands for the *M*_n_ of the PS backbone (i.e., xx × 10^3^ g mol^−1^), BACyy stands for the extent of bromoacylation (i.e., yy%), and Cn stands for the number (n) of carbons in the side chain attached to the PS core in the para position. Several SxxBACyy-Cn samples were synthesized by varying PS molecular weight or using 3-bromopropionyl chloride instead of 6-bromohexanoyl chloride according to a method described in [32].

#### 2.2.3. Synthesis of Bromoalkylated PS (SxxBAKyy-Cn)

The ketone group of SxxBACyy-Cn was reduced using a method described in [32]. A one-neck round-bottom flask equipped with a condenser was charged with SxxBACyy-Cn (1 g) and anhydrous 1,2-dichloroethane (50 mL), and the resulting solution was treated with trifluoroacetic acid (30 mL) and triethylsilane (3.5 mL), heated to 60 °C, and stirred for 30 h. Subsequently, the solution was cooled to room temperature and neutralized by pouring into 2 M aqueous KOH. The obtained mixture was stirred overnight at 60 °C, and the organic phase was separated and poured into excess methanol. The solid precipitate was washed several times with methanol and vacuum-dried at 80 °C to afford bromoalkylated PS in 90% yield. The obtained samples were denoted as SxxBAKyy-Cn, where Sxx stands for the *M*_n_ of the PS backbone (xx × 10^3^ g mol^−1^), BAKyy stands for the content of the bromoalkylated portion (yy%), and Cn stands for the number (n) of carbons in the side chain attached to the PS core in the para position.

#### 2.2.4. Functionalization of Bromoalkylated PS (SxxQAyy-Cn)

A solution of SxxBAKyy-Cn (1 g) in anhydrous DMF (7 mL) prepared at 60 °C was supplemented with excess trimethylamine solution (2 mL), stirred for 16 h, filtered using a 0.45-μm syringe filter, and poured into a flat glass Petri dish. The excess trimethylamine, moisture, and solvent were removed by 48 h drying in a vacuum oven, and the resulting brittle membranes were wetted with deionized water, peeled off, washed with deionized water, and dried at 60 °C in a vacuum oven.

### 2.3. Polymer Characterization

^1^H NMR spectra were recorded on a 400-MHz Bruker AVANCE-III spectrometer. Typically, samples (0.01 g) were dissolved in 0.8 mL of CDCl_3_ or, in the case of functionalized polymers, in the same volume of dimethyl sulfoxide-d6. The assigned peaks were used for polymer structure determination and end-group analysis.

Polymer molecular weight and PDI were determined by gel permeation chromatography (GPC; Waters 2414 refractive index detector) using 0.05 M LiBr in NMP as an eluent. Typically, PS copolymers (6 mg) were dissolved in 0.05 M LiBr in NMP, and the solution was filtered through a 0.45-μm syringe and analyzed by GPC at 40 °C and a flow rate of 1.0 mL min^−1^.

Thermal decomposition behavior was analyzed using a Q50 apparatus (TA Instruments). After 1 h drying at 100 °C, an approximately 7 mg sample was heated at 10 °C min^−1^ from 40 to 600 °C in an atmosphere of N_2_.

Ion-exchange capacity (IEC) was determined using the back-titration method. The membrane fragments in the bromide foam were soaked in 1 M aqueous KOH for 48 h and washed with deionized water to remove the residual KOH. After conversion from the bromide form to the hydroxide form, the functionalized membrane fragments were dried at 80 °C in a vacuum oven for 24 h, weighed (*W*_dry_), and immersed into a 0.01 M standard HCl solution for 48 h to exchange OH^−^ for Cl^−^. The remaining liquid was titrated with 0.05 M NaOH using phenolphthalein as an indicator, and IECs were calculated as

IEC = *C*(*V*_0_ − *V*_x_)/*W*_dry_, (1)

where *V*_0_ and *V*_x_ are the volumes of blank (sample without membranes) and consumed NaOH solutions, respectively, and *C* is the concentration of the NaOH solution.

Transmission electron microscopy (TEM) was used to investigate the hydrophilic–hydrophobic morphology of the ionomers. Cryo-TEM investigations were performed on a Tecnai F20 G2 (FEI Co., USA) with an accelerating voltage of 200 kV. Before observation, the membrane fragments were immersed in a 0.5 M potassium tetrachloroplatinate (II) aqueous solution to exchange the Br^−^ ions for PtCl_4_^2−^ at 40 °C for 48 h, then rinsed with deionized water, and dried in a vacuum oven at 60 °C. The samples were embedded in epoxy resin, sectioned to a thickness of 70 nm with a Leica microtome Ultracut UCT, and finally collected on copper grids.

### 2.4. Ionomer Preparation

The functionalized PS copolymers were fully dissolved in 1-propanol: deionized water (70:30, *w*/*w*) to a content of 5 wt%, and the transparent dispersions were used to fabricate the electrode binder.

### 2.5. Analysis of Ionomer

The ionomer dispersion system was analyzed by dynamic light scattering (DLS) with an ELS-Z Zeta-potential and particle size analyzer (Otsuka Electronics Co., Japan). Each ionomer (20 mg) was diluted with 2-propanol (IPA, 9.98 g). The measurement temperature was fixed at 25 °C, and the measurements were conducted at least six times.

### 2.6. MEA Fabrication

A dispersion of Pt/C (46.7 wt%, Tanaka, Japan) and the ionomer in deionized water and 2-propanol (Honeywell, USA) was prepared by bath sonication and used as the catalyst ink for the anode and the cathode. The ionomer content was fixed at 30 wt%. The commercial AEM was prepared as a 20-μm-thick non-reinforced membrane in the bromide form (Fumasep FAA-3-20, FuMA-Tech Inc., Germany). The catalyst ink was spray-coated on both membrane sides using the catalyst-coated membrane method, and the resulting MEAs were immersed into 1 M aqueous KOH for 4 h to exchange the bromide ions in the ionomer of the catalyst layer and the AEM for hydroxyl ions. After ion exchange was complete, the MEAs were washed several times with Ar-purged deionized water to remove excess OH^−^ ions. The MEA active area equaled 5 cm^2^, and a Pt loading of 0.4 mg_pt_ cm^−2^ was used for both the anode and the cathode. Sigracet 39BC (340 μm, Sigracet SGL Carbon Inc., Germany) was used as the gas diffusion layer (GDL) of each electrode. The catalyst-coated membranes were sandwiched between GDLs and a Teflon gasket. Finally, the MEAs were assembled into single cells. For comparison, an MEA based on a commercial anion-exchange ionomer (Fumion FAA-3-SOLUT-10, 10 wt% in NMP, FuMA-Tech Inc., Germany) was prepared with the same catalyst compositions.

### 2.7. Single-Cell Performance Test

AEMFC performance was tested using a fuel cell test station (CNL energy, Korea). H_2_ (0.2 L min^−1^) and O_2_ (0.4 L min^−1^) with a relative humidity (RH) of 100% were fed with no backpressure at the anode and cathode, respectively. After cell activation, the current–voltage polarization curves of MEAs were recorded by stepwise loading of the cell current at an operating temperature of 60 °C. Polarization curves of each single cell were obtained twice to confirm constant performance. Electrochemical impedance spectroscopy (EIS) analysis was performed using an impedance analysis device (HCP-803, Biologic, France) in the frequency range of 50 kHz to 100 mHz. Single-cell durability tests were conducted at a constant current density of 200 mA cm^−2^.

## 3. Results and Discussion

### 3.1. Polymer Synthesis and Structural Analysis

**Scheme 1:**
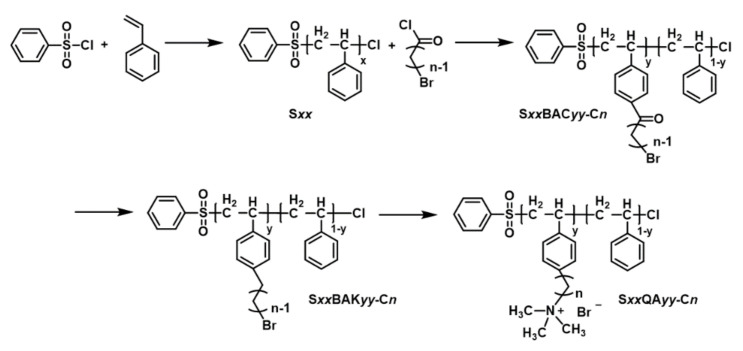
Synthesis of polystyrene (PS)-based hydroxide-ion-conducting ionomers.

The synthesis of the PS-based hydroxide-ion-conducting ionomer is illustrated in Scheme 1. Both the *M*_n_ and PDI of the PS backbone increased with increasing styrene:benzenesulfonyl chloride ratio (Table 1), which limited the termination of the coupling reaction. The PS-based anion-exchange ionomer functionalized with quaternary alkylammonium groups was prepared by the acylation of PS at the para position of the benzene ring followed by C=O group reduction to form bromoalkylated PS and quaternization.

**Figure 1:**
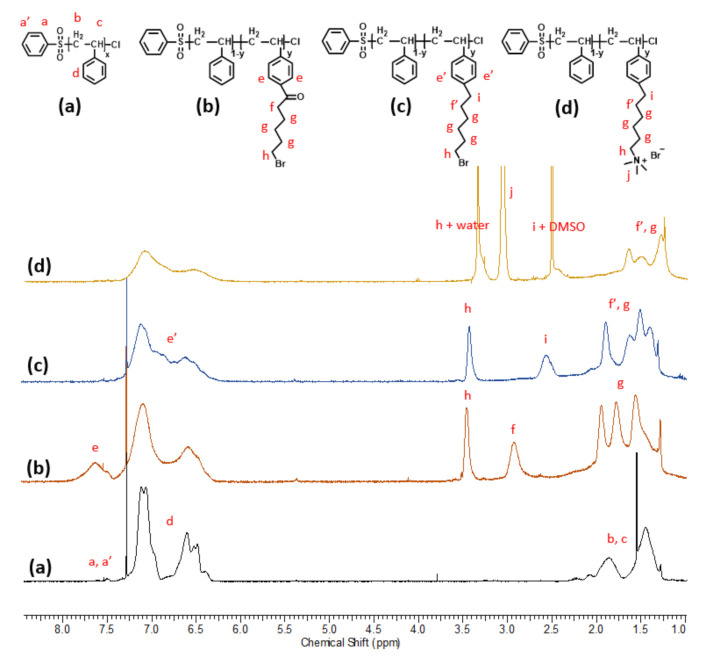
^1^H NMR spectra of (**a**) PS (Sxx), (**b**) bromoacylated PS (SxxBACyy-Cn), (**c**) Bromoalkylated PS (SxxBAKyy-Cn), and (**d**) quaternized PS (SxxQAyy-Cn) samples.

^1^H NMR spectra corresponding to the products of each synthesis step (Figure 1) provided structural information and were used for the end-group-analysis-based determination of molecular weight. The presence of the PS backbone was confirmed by the signals of the benzenesulfonyl end groups of the polymer and those of styrene units (Figure 1a). The aromatic protons adjacent to SO_2_ groups (*H_a_* and *H_a’_*) resonated at 7.66–7.40 ppm, while signals at 7.27–6.25 ppm (*H_d_*) and 2.03–1.18 ppm (*H_b_*, *H_c_*) were attributed to the protons in PS repeating units. The *M*_n_ of PS was determined as 10,576 g mol^−1^ from the ratio of the integral intensity of the *H_a_* + *H_a’_* peak to that of the *H_d_* peak (Figure S1) and was close to the value of 9735 g mol^−1^ obtained by GPC.

Acylation was performed at the p-position of styrene to obtain bromoacylated copolymers [33] that were subsequently reduced and reacted with trimethylamine to replace Br with N(CH_3_)_3_^+^ groups, with the quaternization degree fixed at 30% (Figure 1b). The spectra of bromohexanoyl-modified polymers featured signals of benzene ring protons adjacent to the ketone group (*H_e_*) at 7.85–7.45 ppm and peaks of the bromoalkyl chain protons at 3.51–3.34 ppm (*H_h_*), 3.02–2.81 ppm (*H_f_*), and 2.02–1.25 ppm (*H_g_*).

As the electron-withdrawing ketone groups could accelerate the degradation of quaternary ammonium units under alkaline conditions, they were reduced to CH_2_ groups, as confirmed by the appearance of a new peak at 2.69–2.43 ppm (*H_i_*) and the shift of *H_e’_* and *H_f’_* peaks (Figure 1c).

Anion-conducting cationic groups were introduced through homogeneous amination [34] of bromoalkylated PS with trimethylamine in an organic solvent, and quaternization was confirmed by the appearance of a methyl peak at 3.13–2.97 ppm (*H_j_*) (Figure 1d).

Several polymers (SxxQAyy-Cn) were synthesized by the abovementioned method using different benzenesulfonyl chloride:styrene ratios (Figure S1) and acylation reagents (Figure S2), with the reaction parameters and molecular weights of these polymers listed in Table 1.

### 3.2. IEC and Thermal Decomposition Behavior

The brittleness of PS-based anion-exchange ionomers complicated the formation of membranes in the absence of crosslinking or blending (Figure S3). Hence, the properties of ionomers synthesized under various conditions were compared in terms of the IECs of the corresponding membrane fragments. IECs, representing the amount of functional groups capable of conducting hydroxide ions per dry polymer weight (mmol g^−1^), were measured by an acid–base back-titration method. At a constant degree of functionalization (DF = 30%), IECs of ~1.45 mmol g^−1^ were obtained for samples irrespective of the molecular weight of the polymer backbone. In addition, the IEC of the S29-main-chain polymer with a C3 alkyl side chain (1.74 mmol g^−1^) exceeded that of the polymer with the longer C6 side chain. As the length of the flexible alkyl ammonium side chain increased, the IEC value decreased because of the increased hydrophobicity of the alkyl side chain [35]. In a previously reported study, the nature and location of cations in AEMs had a significant effect on their IEC and ion conductivity values. This effect was due to the difference in ion transport in the hydrophilic–hydrophobic phase separation along the side chain length according to whether the AEMs were functionalized with quaternary ammonium or quaternary piperidinium [17]. In agreement with these findings, the position of quaternary ammonium units along the side chain length affected the IECs of our samples.

**Figure 2:**
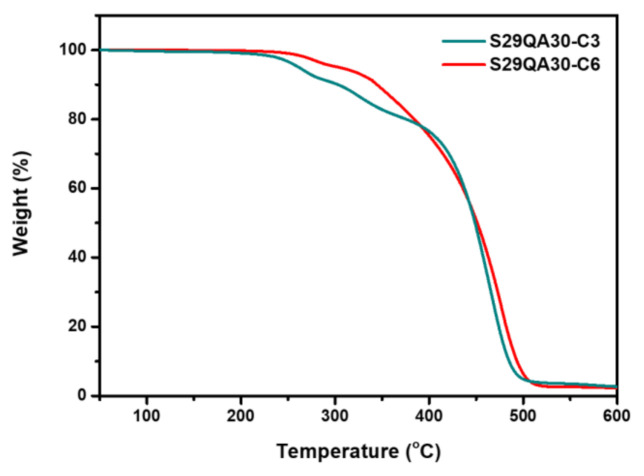
Thermogravimetric analysis (TGA) curves of bromide-form S29QA30-C3 and S29QA30-C6.

The thermal stability of bromide-form ionomers was evaluated by thermogravimetric analysis (TGA) under N_2_ (Figure 2), and two decomposition steps were observed. The first weight loss (180–380 °C) was ascribed to the decomposition of functional groups, and the second one (>400 °C) was ascribed to PS backbone degradation. These results indicate the good thermal stability of the ionomers having quaternary ammonium groups. Moreover, all polymers are suitable for use in AEMFCs, as these cells are commonly operated below 100 °C.

### 3.3. Morphology

**Figure 3:**
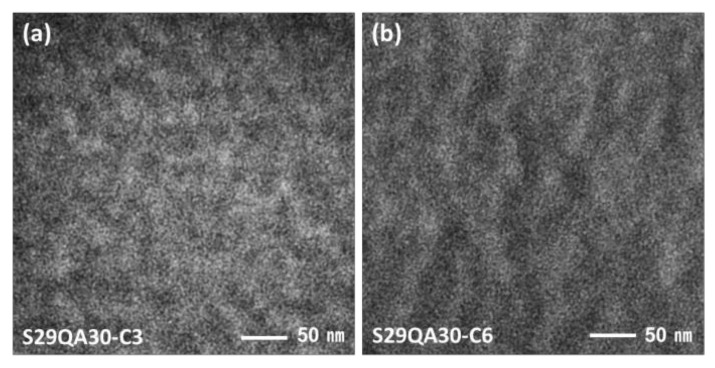
TEM images of (**a**) S29QA30-C3 and (**b**) S29QA30-C6.

S29QA30-C3 and S29QA30-C6 were ion-exchanged from Br^−^ ions to PtCl_4_^2−^ ions for TEM imaging (Figure 3a,b, respectively), wherein, the dark regions correspond to hydrophilic domains, which are stained quaternary ammonium groups, while the bright regions represent the hydrophobic domains composed of the polymer backbone and alkyl side chain. As shown in Figure 3, both samples showed distinct hydrophilic–hydrophobic morphology. S29QA30-C6 exhibited more continuous phase-separated morphology than S29QA30-C3. The width of the domains of aggregated ionic clusters was measured to be 12–17 nm for S29QA30-C3 and 25–36 nm for S29QA30-C6. These results suggest that the long alkyl chain between the PS backbone and quaternary ammonium groups developed the formation of longer, wider ionic clusters.

### 3.4. Dispersion Property

**Figure 4:**
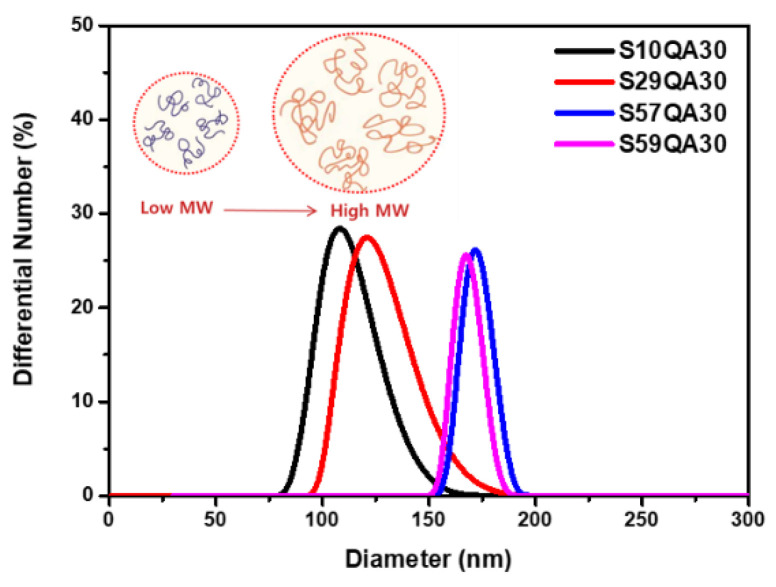
Dynamic light scattering (DLS) analysis according to the molecular weight of ionomers.

The ionomer dispersions were observed using DLS, and Figure 4 compares the agglomerated polymer sizes in the dispersions, which were diluted to 0.01 wt% in IPA, which corresponds to the solvent of the catalyst ink. The PS-based ionomers agglomerated in the range of 80–200 nm according to the four different molecular weights. Increasing the polymer molecular weight means lengthening the linear chains. Because longer polymers tend to become more entangled, their tendency to agglomerate increases with their molecular weight, which in turn increases the size of the agglomerates.

### 3.5. AEMFC Performance

#### 3.5.1. Effect of PS Backbone Length of the Sxxqa30-C6 Ionomer

**Figure 5:**
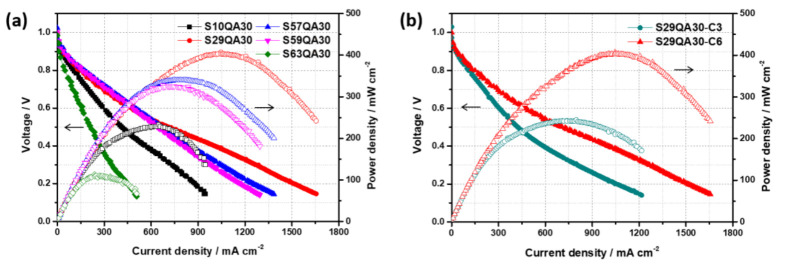
Effects of the (**a**) molecular weight and (**b**) alkyl chain length of the ionomers on the polarization curves. Test conditions: Fumasep FAA-3-20 membrane, Pt loading = 0.4 mg_Pt_ cm^−2^ for both anode and cathode, cell temperature = 60 °C, H_2_ flow rate = 0.2 L min^−1^, O_2_ flow rate = 0.4 L min^−1^ (100% RH), no backpressure.

As shown in Figure 5a, the AEMFC performance was measured for the five different ionomers with PS backbones of different lengths. The highest-molecular-weight ionomer (S63QA30-C6) was partly lumpy and afforded an opaque dispersion (Figure S4), i.e., it was difficult to completely disperse in the alcohol:water mixture. Therefore, the MEA with S63QA30-C6 exhibited the lowest current density of 160 mA cm^−2^ at 0.6 V owing to the highest degree of chain entanglement induced by this non-uniform dispersion. On the other hand, the MEA with the S10QA30-C6 ionomer, which had a lower degree of chain entanglement, showed poor physical binder performance and insufficient anion conduction [36], which resulted in low current density of 289 mA cm^−2^ at 0.6 V. However, the other three ionomers performed significantly better than S10QA30-C6 and S63QA30-C6 overall because of their higher ion conduction and better binder properties in the electrode layer. In addition, the strong chain entanglement of S57QA30-C6 and S59QA30-C6 resulted in slightly better performance than that of S29QA30-C6 up to the ohmic region (current density = 500 mA cm^−2^ for S57QA30-C6 and S59QA30-C6 and 483 mA cm^−2^ for S29QA30-C6), while slightly worse performance was observed in the mass transport region. This result can be interpreted as the large particles in the dispersions with the high molecular weight hindering the mass transport of the reactant owing to the agglomeration of the catalyst and ionomer in the electrode.

The effects of the molecular weight of the ionomers were further probed by EIS analysis of cells at 0.4 V, as shown in Figure S5. The single-cell performance was found to correlate with the ionomer type. The smallest ohmic resistance of 0.0897 Ω cm^2^ was observed for S29QA30-C6, while values of 0.11–0.25 Ω cm^2^ were obtained for the other samples. In particular, S63QA30-C6, which exhibited the largest charge transfer resistance, demonstrated mass transport limitations, even in the polarization curve (Figure 5a). The resistance measured at 0.4 V and the high current density mainly includes the effects of the polymer electrolyte membrane and the ionomer, which may have been due to flooding at the anode, drying at the cathode, or both [37]. Therefore, the electrode fabricated using S29QA30-C6 was superior to that produced using the higher-molecular-weight electrode binder, as the former ionomer provided a well-connected ion conduction path in the catalyst layer and facilitated the discharge of supplied gas and generated water, which was conducive to the electrochemical reaction.

According to our previous study [36], cell performance is strongly dependent on the molecular weight of the binder, with higher-molecular-weight binders promoting agglomeration in the electrode and thus lowering cell performance. This finding seemed to apply to the present study.

#### 3.5.2. Effect of Side Chain Length

Figure 5b shows the polarization curves of S29QA30-C3- and S29QA30-C6-containing AEMFCs recorded at maximum power densities of 250 and 407 mW cm^−2^, respectively, revealing that cell performance decreased with the decreasing length of the alkyl side chain. As the change in the alkyl side chain length at constant DF in turn affected the IEC (Table 1), the above performance difference was related to the problem of water management in the electrodes, the complexity of which is an issue with AEMFCs [38,39]. Generally, high IECs result in high water uptake and may therefore cause cell flooding, in which case the discharge of the generated water does not proceed smoothly, and the temporarily increased water content reduces cell performance by hindering H_2_ transport and access to catalytically active sites. To improve water management, one can change the hydrophilicity–hydrophobicity/porosity of the GDL or the gas flow rate/humidity [3,40,41,42].

Another problem is considered to be the decreasing probability that Pt/C as the HOR catalyst adsorbs onto the ionomer with the increasing distance between the phenyl group of styrene and quaternary ammonium. The space between the main chain leading to the aryl-ether-free C–C bond and the functional group must be examined to resolve this phenyl adsorption problem. Therefore, this study employs a different approach from that in previously reported studies on the phenyl adsorption of polyaromatic-based ionomers [23,24,43]. We demonstrated that the alkyl side chains directly affected the AEMFC performance while maintaining the backbone of the aryl-ether-free PS ionomer without changing the polymer backbone structure. However, it is difficult to elucidate which of the two possible approaches is advantageous, and further research is required.

#### 3.5.3. Short-Term Durability

**Figure 6:**
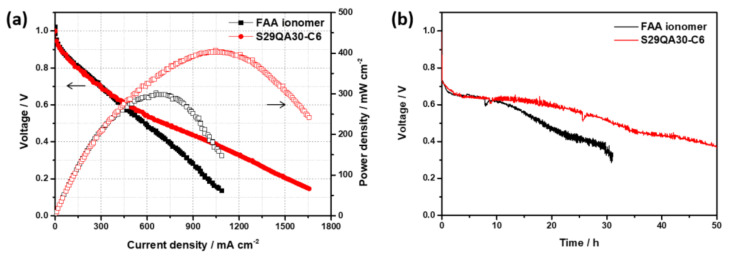
Comparison of commercial ionomer (Fumion FAA-3 solution)- and S29QA30-C6-ionomer-based MEAs: (**a**) polarization curves and (**b**) results of the short-term durability test at a current density of 200 mA cm^−2^. Test conditions: Fumasep FAA-3-20 membrane, Pt loading = 0.4 mg_Pt_ cm^−2^ for both anode and cathode, cell temperature = 60 °C, H_2_ flow rate = 0.2 L min^−1^, O_2_ flow rate = 0.4 L min^−1^ (100% RH), no backpressure.

Herein, S29QA30-C6 (which exhibited the best initial cell performance) was selected for the evaluation of fuel cell durability, and an MEA with the FAA ionomer was used as a reference. As we measured the AEMFC performance using the commercial FAA ionomer and membrane, the maximum power density was 298 mW cm^−2^ in Figure 6a. In a recently reported investigation [44], a commercial MEA investigated by another research group exhibited a maximum power density of 275 mW cm^−2^ with the same materials under similar operation conditions. Therefore, it is reasonable to compare the performance of the present ionomers with that of the FAA ionomer using the commercial FAA membrane in this study. Both MEAs were assembled using the commercial FAA-3-20 membrane and differed only in the electrode ionomer. As shown in Figure 6a, the two MEAs showed similar performances in the activation region, while the S29QA30-C6 MEA featured superior performance in the ohmic region. At 0.85 V, both MEAs had similar EIS characteristics, while at 0.4 V, the ohmic and charge transfer resistances of the FAA ionomer significantly increased, in line with the fact that the corresponding polarization curve showed mass transport limitations in the low-voltage region (Figure S6). The increase in resistance at the low voltage of 0.4 V was due to the increase in ohmic resistance caused by the water transport mismatch of the membrane/electrode interface, which, in turn, was a result of complex water management [45]. Compared to MEAs assembled with commercial polyaromatic-compound-based membranes and ionomers, the MEA with S29QA30-C6 had a lower resistance due to the improved water management of the electrode.

Finally, the S29QA30-C6-based MEA and the FAA-ionomer-based reference MEA were subjected to short-term durability testing at a constant current density (200 mA cm^−2^) and an operation temperature of 60 °C using fully humidified H_2_ and O_2_ and no backpressure (Figure 6b). The test was performed without MEA replenishment. The onset potentials of S29QA30-C6 and the FAA binder were determined to be similar, namely, 0.748 and 0.738 V, respectively. However, the deterioration of the FAA binder accelerated after 10 h, with an average decay rate of 13.6 mV h^−1^ over 31 h. On the other hand, S29QA30-C6 showed a lower average voltage decay rate of 7.5 mV h^−1^ over 50 h. Thus, the polyaromatic-compound-based FAA-3-based MEA containing the aryl ether bond showed lower durability than the S29QA30-C6 MEA-based, which greatly mitigated the degradation of the performance. Coupled with the higher overall performance of the S29QA30-C6 MEA, these results are thus concluded to pave the way to the fabrication of AEMs with various polymer structures for improving the performance of AEMFCs.

## 4. Conclusions

Hydroxide-ion-conducting electrode ionomers based on quaternary-ammonium-group-functionalized PS with controlled molecular weight and side chain length were prepared by radical polymerization of styrene followed by acylation, reduction, and quaternization. The length of the polymer backbone directly affected AEMFC performance, and an appropriate IEC and functionality were related to the hydration of the electrode layer, which highlighted its importance. The extent of degradation during short-term operation was closely related to the polymer structure of the electrode binder, and MEAs fabricated using PS-based ionomers showed better durability than those fabricated using a commercial polyaromatic-compound-based binder solution.

## Figures and Tables

**Table 1:** Properties of as-synthesized copolymers.

Sample	Reaction Parameter for PS Backbone			Molecular Weight of PS Backbone			IEC ^e^ (mmol g^−1^)
	Ratio ^a^	Reaction Time (h)	Yield (%)	*M*_n_ (g mol^−1^) ^b^	*M*_n_ (g mol^−1^) ^c^	PDI ^d^	
S10QA30-C6	65	24	22.6	10,576	9735	1.56	–
S29QA30-C3	300	24	18.1	–	28,893	1.83	1.74 ± 0.06
S29QA30-C6	300	24	18.1	–	28,893	1.83	1.47 ± 0.05
S57QA30-C6	700	24	13.8	–	56,950	2.01	1.45 ± 0.08
S59QA30-C6	700	24 ^f^	13.6	–	58,931	2.69	1.45 ± 0.05
S63QA30-C6	1000	24	14.9	–	63,067	1.96	1.44 ± 0.03

^a^, Styrene:benzenesulfonyl chloride ratio; ^b^, calculated from the integral intensities of ^1^H NMR signals; ^c^, determined by gel permeation chromatography (GPC); ^d^, polydispersity index (*M*_w_/*M*_n_); and ^e^, titrated values. ^f^, S59 was synthesized using less synthetic solvent, namely, 30% relative to that used for S57.

## Data Availability

The data presented in this study are available on request from the corresponding author.

## Supplementary Materials

The following are available online at https://www.mdpi.com/2073-4360/13/5/690/s1, Figure S1: ^1^H NMR spectra of ionomers with various polystyrene backbones (S*xx*), where S stands for polystyrene, and *xx* stands for the number average molecular weight (*M*_n_ = *xx* × 10^3^ g mol^−1^); Figure S2: ^1^H NMR spectra ((a–c) in CDCl_3_ and (d) in DMSO-d_6_) of compounds produced during the synthesis of S29QA30-C3. (a) S29, (b) S29BAC30-C3, (c) S29BAK30-C3, and (d) S29QA30-C3; Figure S3: Images of bromide-form S29QA30-C6 membrane fragments; Figure S4: Images of (a) S29A30-C6 and (b) S63QA30-C6 ionomer dispersions; Figure S5: Effects of ionomer molecular weight on EIS spectra measured by 0.4 V; Figure S6: EIS spectra measured at 0.85 V and 0.4 V for comparison between MEAs based on the commercial ionomer (Fumion FAA-3 solution) and S29QA30-C6 ionomer.
