# Supplementary material for: Polystyrene-Based Hydroxide-Ion-Conducting Ionomer: Binder Characteristics and Performance in Anion-Exchange Membrane Fuel Cells
Source: Polymers (Basel). 2021 Feb 25;13(5):690. doi: 10.3390/polym13050690 (PMC7956690; doi:10.3390/polym13050690)
Supplement: Supplementary file 1 [file polymers-13-00690-s001.pdf]

Supplementary materials

Polystyrene-Based Hydroxide-Ion-Conducting Ionomer: Binder Characteristics and Performance in Anion-Exchange Membrane Fuel Cells

Ji Eon Chae[1,2], So Young Lee[1], Sung Jong Yoo[1], Jin Young Kim[1], Jong Hyun Jang[1], Hee-Young Park[1], Hyun Seo Park[1], Bora Seo[1], Dirk Henkensmeier[1], Kwang Ho Song[2,*], Hyoung-Juhn Kim[1,*]

[1]Center for Hydrogen and Fuel Cell Research, Korea Institute of Science and Technology (KIST), Hwarang-ro 14-gil 5, Seongbuk-gu, Seoul 02792, Republic of Korea; 218016@kist.re.kr (J.E.C.); sylee5406@kist.re.kr (S.Y.L.); ysj@kist.re.kr (S.J.Y.); jinykim@kist.re.kr (J.Y.K.); jhjang@kist.re.kr (J.H.J.); parkhy@kist.re.kr (H.-Y.P.); hspark@kist.re.kr (H.S.P.); brseo@kist.re.kr (B.S.); henkensmeier@kist.re.kr (D.H.)

[2]Department of Chemical and Biological Engineering, Korea University, Anam-ro 145, Seongbuk-gu, Seoul 02841, Republic of Korea

*Correspondence: hjkim25@kist.re.kr; Tel.: +82-2-958-5299 (H.-J. K.); khsong@korea.ac.kr; Tel.: +82-2-3290-3307 (K.H.S.)

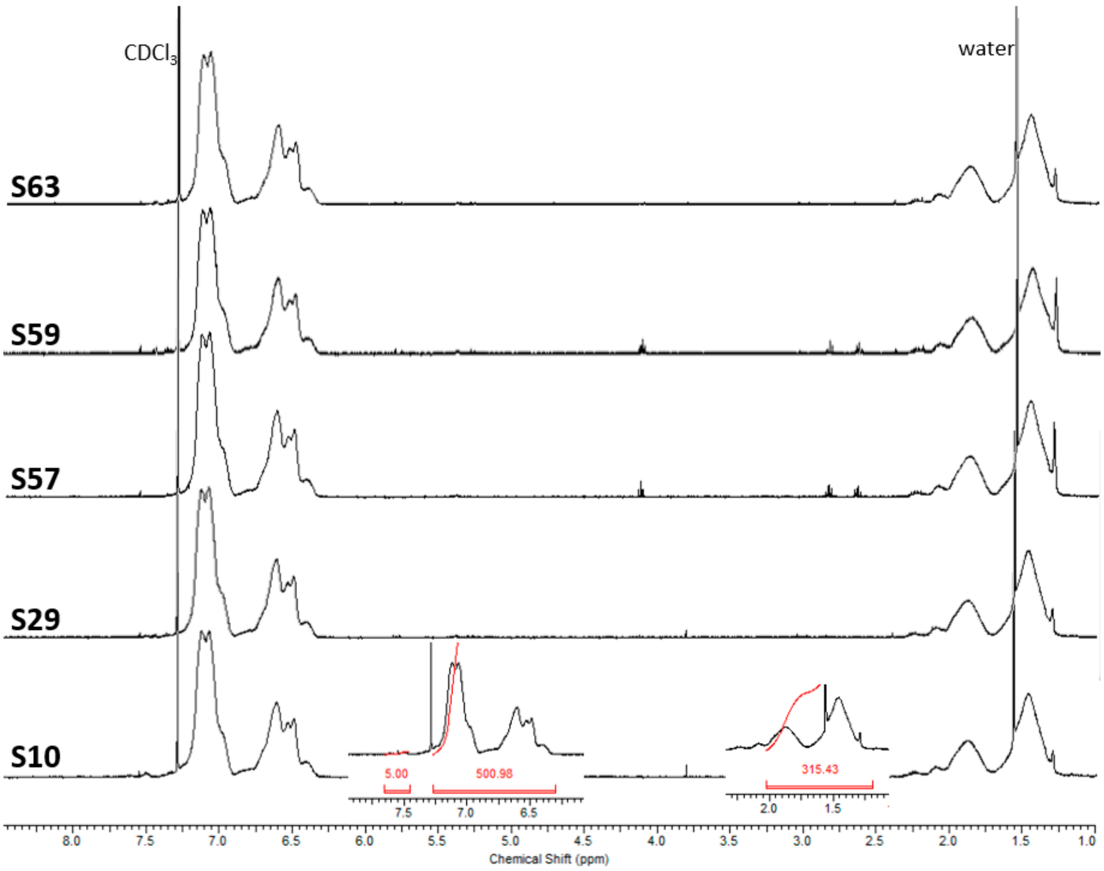

Figure S1. ^{1}H NMR spectra of ionomers with various polystyrene backbones (S*xx*), where S stands for pol ystyrene and *xx* stands for the number average molecular weight (M_n = *xx* × 10^3 g mol^{-1}).

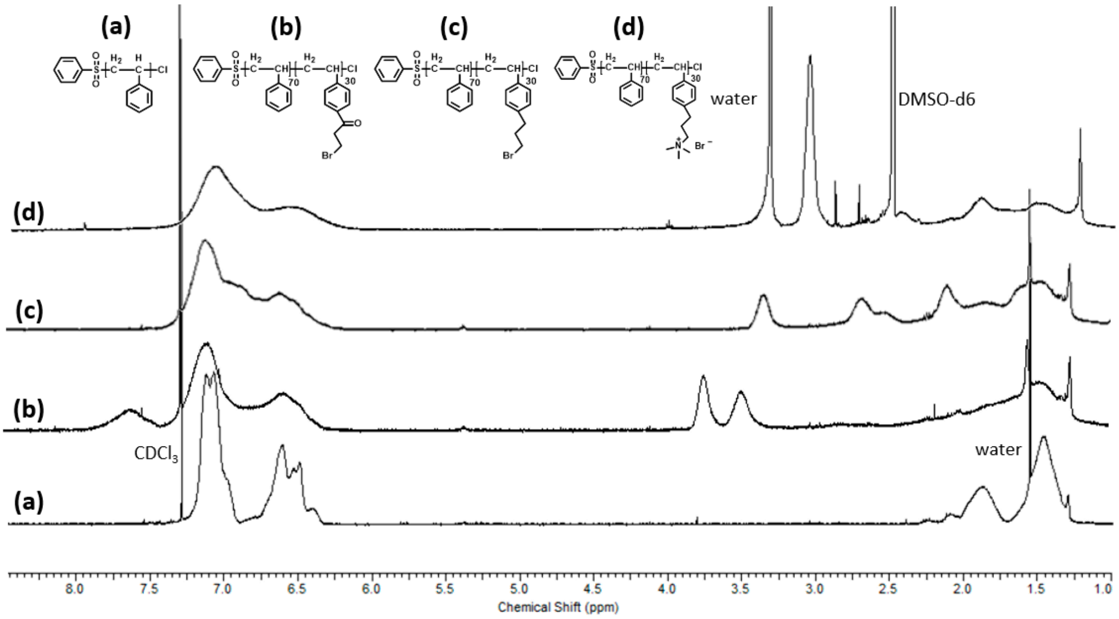

Figure S2. ^{1}H NMR spectra ((**a**–**c**) in $CDCl_3$ and (**d**) in DMSO-d_6) of compounds produced during the synt hesis of S29QA30-C3. (**a**) S29, (**b**) S29BAC30-C3, (**c**) S29BAK30-C3, (**d**) S29QA30-C3.

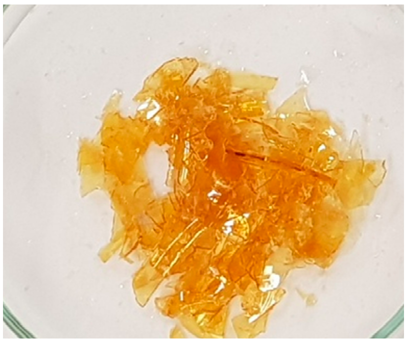

Figure S3. Images of bromide-form S29QA30-C6 membrane fragments.

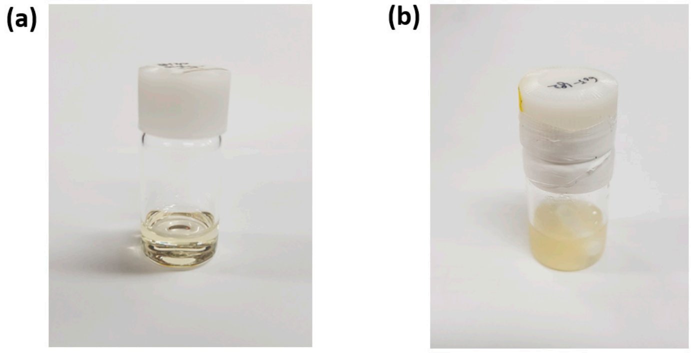

Figure. S4. Images of (**a**) S29A30-C6 and (**b**) S63QA30-C6 ionomer dispersions.

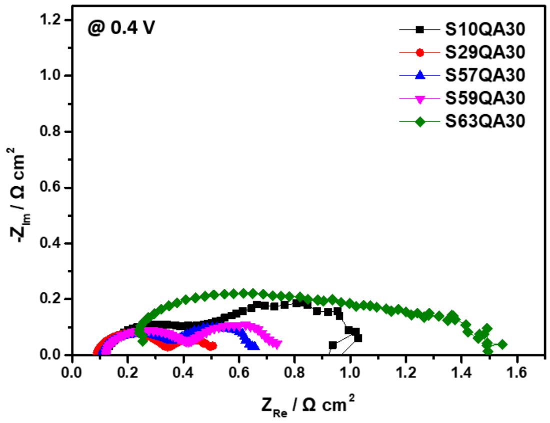

Figure S5. Effects of ionomer molecular weight on EIS spectra measured by 0.4 V.

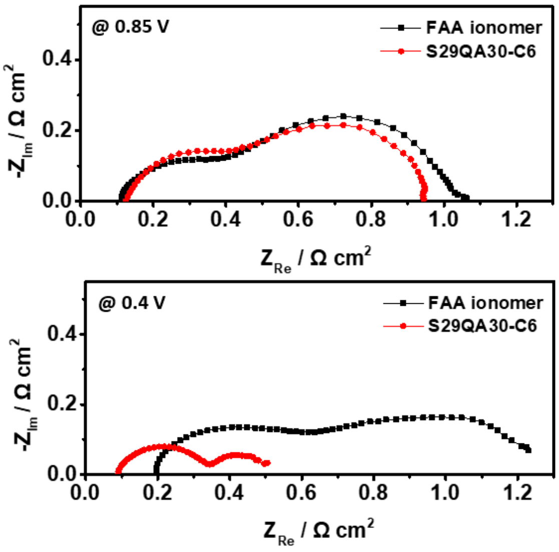

Figure S6. EIS spectra measured at 0.85 V and 0.4 V for comparison between MEAs based on the commer cial ionomer (Fumion FAA-3 solution) and S29QA30-C6 ionomer.
